# Supplementary material for: A PDLP-NHL3 complex integrates plasmodesmal immune signaling cascades
Source: Proc Natl Acad Sci U S A. 2023 Apr 17;120(17):e2216397120. doi: 10.1073/pnas.2216397120 (PMC10151459; doi:10.1073/pnas.2216397120)
Supplement: Supplementary file 1 — Appendix 01 (PDF) [file pnas.2216397120.sapp.pdf]

PNAS

**Supporting Information for**

# A PDLP-NHL3 complex integrates plasmodesmal immune signaling cascades

Estee E. Tee[1], Matthew G. Johnston[1], Diana Papp[1,2], Christine Faulkner[1]

[1]Cell and Developmental Biology, John Innes Centre, NR4 7UH Norwich, United Kingdom

[2]Present address: Ludwig Institute for Cancer Research, Oxford University, Oxford, United Kingdom

**Email:** christine.faulkner@jic.ac.uk

**This PDF file includes:**

Extended Material and Methods

Figures S1 to S11

Tables S1

SI References

## Extended Material and Methods

*Microprojectile bombardment assays*

Leaves of 5- to 6-week-old *Arabidopsis* were used for assaying plasmodesmal permeability, with a minimum of four leaves used per genotype and treatment. Bombardment assays were performed using 1 nm gold particles (BioRad) coated with pB7WG2.0-GFP and pB7WG2.0-$RFP_{ER}$ using a Biolistic PDS-1000/HE particle delivery system (BioRad). Treatments [mock ($dH_2O$), chitin (500 µg/mL), flg22 (100 nM), SA (100 µM), or $H_2O_2$ (10 mM)] were syringe infiltrated two hours post-bombardment. To visualize bombardments, GFP was excited with a 488 nm argon laser and collected at 505-530 nm while mRFP was excited with a 561 nm DPSS laser and collected at 600-640 nm. The number of cells showing GFP was normalized to the mean of the mock-treated data within a genotype to produce the relative spread of GFP, and n stated in each legend is bombardment sites collected per genotype/treatment, with the exception of when comparing absolute number of cells showing GFP, where the number was not normalized as no treatment was used.

*Generation of CRISPR-Cas9* cml41 *null mutant*

A construct for CRISPR-Cas9 gene editing was assembled as described (1). sgRNAs targeting *CML41* were designed and assembled by PCR and Golden Gate cloning with a *Ubi10 promoter::Cas9::Nos* terminator cassette and a bialaphos resistance gene (*bar*) in a binary vector for plant expression (Fig. S3A). The target sgRNA binding site in the *CML41* gene resulted in a 193 bp deletion (Fig. S3A) and introduced a premature stop codon. The *cml41* deletion was confirmed in candidate knockouts by genotyping (Fig. S3B; using primers GACATCTCCATCCATGAGAAACC and TGGCAAATGTTCAGCAGAGC), and *CML41* transcript levels were assessed by RT-PCR. 10-day old seedlings were grown on Murashige and Skoog 1% sucrose 0.8% agar plates under 16 h light at 22 °C. Total RNA was isolated using an RNeasy® Plant Mini Kit (Qiagen), according to the manufacturer's instructions. RNA was treated with a DNase treatment using the 'Rigorous DNase treatment' protocol from the TURBO DNA-*free*™ Kit (Invitrogen). cDNA was synthesized from 1 µg of RNA using the High-Capacity cDNA Reverse Transcription Kit with RNase Inhibitor (Thermo Fisher Scientific). PCR amplification using a 1:10 dilution template from the synthesized cDNA, was performed with GoTaq Green Master Mix (Promega) using either 25 amplification cycles for the

housekeeper gene *GAPC2* as a control (using primers TCGGAAGAATCGGTCGTTTGG and TGTATGTCATGTACTCGGTGG) or 35 amplification cycles for *CML41* (using primers TGATGACAAGAAGAGATCTTTACGG and CATCTCAAACGCTGTCTTCAA). RT-PCR analysis confirmed *cml41* mutants did not produce full length *CML41* transcript (Fig. S3C). Loss of flg22-induced plasmodesmata closure confirmed the *cml41* mutant phenotype (Fig. S3D).

*Cloning of plant expression constructs*

For transient expression of genes in this study (*PDLP5*, *NHL3*, *LTI6b*, *PDLP1*) coding sequences were cloned with internal BpiI/BsaI/Esp3I sites removed for Golden Gate assembly. All genes were fused with an epitope tag (eGFP, mCherry, $YFP^N$, $YFP^C$) and assembled for *in planta* expression with a 35S CaMV promoter (*p35S*) and a CaMV 35S or *heat shock protein 18.2* (*HSP*) terminator. As an exception, *LTI6b* was fused with the plant *ACTIN2* promoter (*pACT2*) for FRET-FLIM experiments (2). The BiFC YFP secreted 'SP' constructs had the LYM2 signal peptide fused to the designated YFP part.

*Transient expression in N. benthamiana*

*Agrobacterium tumefaciens* GV3101 was transformed with constructs and cultured overnight at 28 °C with appropriate antibiotics. Leaves of 4-week-old *N. benthamiana* plants were syringe-infiltrated with cultures resuspended in infiltration buffer [0.01 M 2-(N-morpholino)ethanesulfonic acid pH 5.6, 0.01 M $MgCl_2$, 0.01 M acetosyringone]. All constructs were co-infiltrated with an *Agrobacterium* strain carrying the p19 silencing suppressor, with each infiltrated at 0.5 $OD_{600nm}$ with the exception of *35S::LTI6b-mCherry* (0.2 $OD_{600nm}$). Tissue for subsequent experiments was collected or imaged two days post infiltration.

*Co-immunoprecipitation*

*N. benthamiana* leaf discs of transiently transformed tissue were frozen and homogenized. Proteins were extracted in an immunoprecipitation (IP) buffer [50 mM Tris-HCl pH 7.5, 150 mM NaCl, 1 mM EDTA, 0.5% NP40 IPEGAL® CA-630 (Sigma), 10% glycerol, protease inhibitor cocktail (Sigma) 1:100, phosphatase inhibitor (Sigma) 1:200, 1 mM $Na_2MoO_4 \times 2H_2O$, 1 mM NaF, 1.5 mM activated $Na_3VO_4$, 5 mM dithiothreitol, 1 mM PMSF]. Protein extracts were co-immunoprecipitated with RFP-Trap magnetic agarose affinity beads (Chromotek) for two hours at 4 °C with gentle agitation. Beads were washed four

times with IP buffer and proteins released by heating at 70 °C in an SDS sample loading buffer containing 2-Mercaptoethanol. Proteins were separated by SDS-PAGE and transferred to an Immuno-blot® PVDF membrane.

*Extended statistical analyses*

For microprojectile bombardment assays, data were analyzed by bootstrap method (*medianBootstrap,* (3)). For multiple comparisons of the absolute number of cells showing GFP, data were analyzed by the bootstrap method (*medianBootstraps2*, (4)). For callose quantifications, FRET-FLIM analysis, rosette area measurements, and *Botrytis cinerea* lesion area measurements, a linear mixed-effects model (with independent factors specified in figure legends) was applied using the R package, *lmerTest*. For multiple images taken for a given biological replicate (*i.e.* callose quantifications and FRET-FLIM), the random effect was 'biological replicate'. For the *Botrytis cinerea* infection assay, the random effect was ''repeat'. ANOVAs specified are ANOVA Satterthwaite's Method, with significant differences between factors (denoted in figure legends) determined by post hoc Tukey HSD using the R package, *emmeans.*

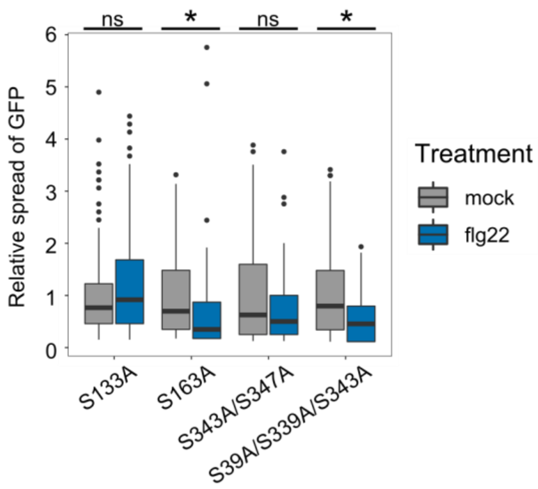


**Fig. S1 RBOHD phosphorylation is required for flg22-induced plasmodesmata closure**

Relative GFP movement to neighboring cells from microprojectile bombardment, with movement into neighboring cells reduced by flg22 in the *rbohD* mutant complemented with $RBOHD_{S163A}$ and $RBOHD_{S39A/S339A/S343A}$, but not in the *rbohD* mutant when complemented with $RBOHD_{S133A}$ or $RBOHD_{S343A/S347A}$ ($n \geq 107$ bombardment sites). Asterisks indicate statistical significance compared to the mock treatment within a genotype: *$p$<0.05.

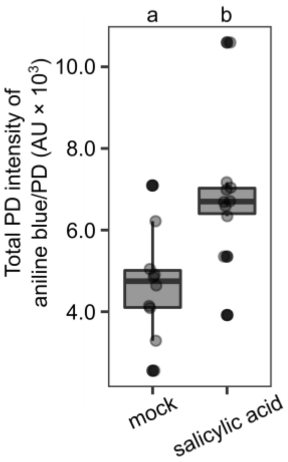


**Fig. S2 Salicylic acid induces plasmodesmata associated callose deposition within 30 minutes**

Fluorescence quantification of aniline blue stained plasmodesmata (PD)-associated callose denoted by arbitrary units (AU) using automated image analysis. 30 minutes post salicylic acid treatment increases fluorescence of callose in Col-0 *Arabidopsis*. Two z-stack image series for five biological replicates were taken per treatment. Independent factor 'Treatment' was determined as significant (ANOVA: $F = 11.7$, $df = 1$, $p<0.005$), with significant differences determined by a post hoc Tukey HSD, denoted by a and b, $p<0.005$.

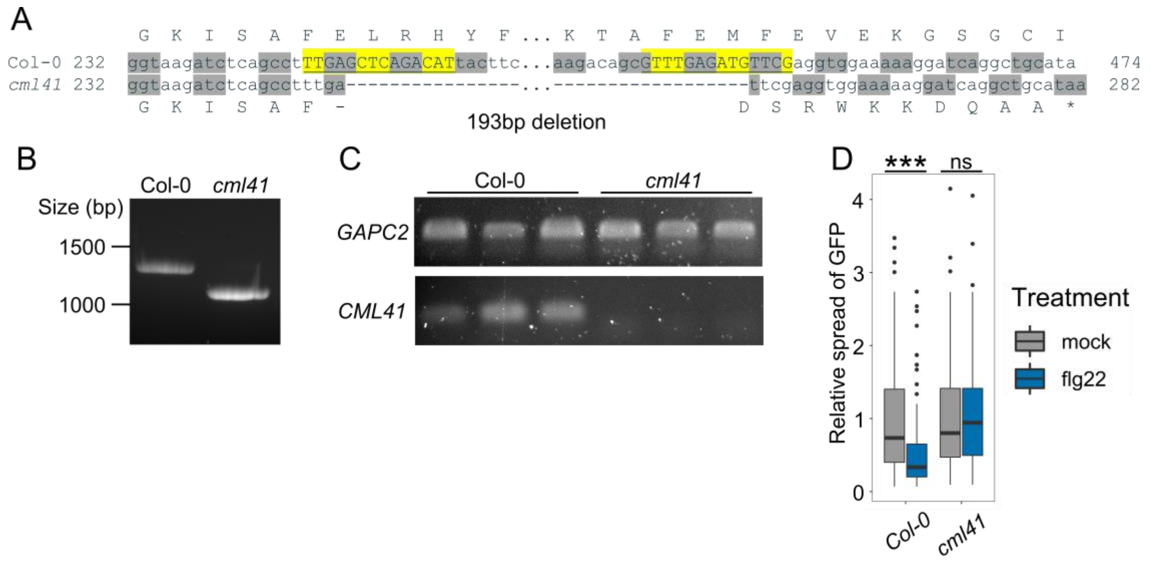


**Fig. S3 Generation of a *cml41* null mutant by CRISPR-Cas9 gene editing**

(A) Target sgRNA binding sites (highlighted in yellow with sequences underlined) in the coding sequence of *CML41* with predicted gene editing introducing amino acid changes and a premature stop codon. (B) A *cml41* null mutant was confirmed by detection of the gene deletion by PCR. (C) No *CML41* transcript was detected in *cml41* by RT-PCR analysis with *GAPC2* expression included as a control. (D) flg22 reduces GFP movement into neighboring cells in Col-0 but not in *cml41* (n≥98 bombardment sites). Data collected from four to seven biological replicates was analyzed by bootstrapping with asterisks indicating statistical significance compared to the mock treatment within a genotype: ****p*<0.001.

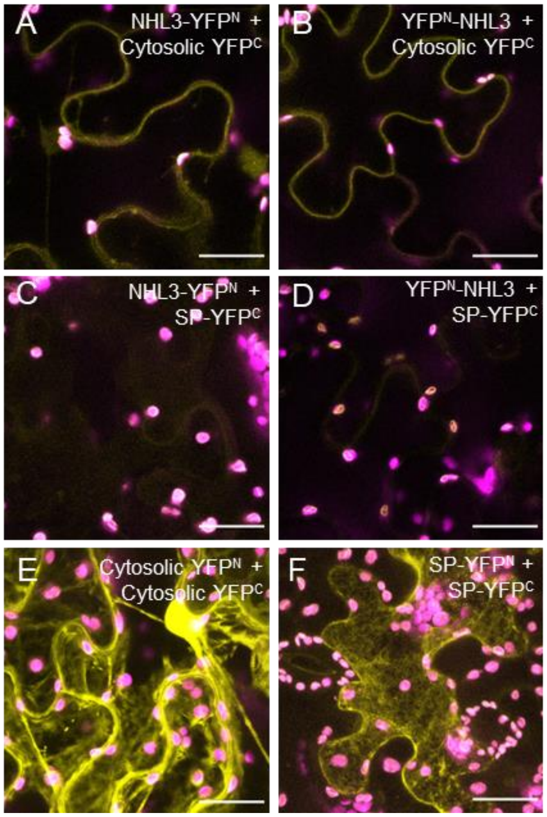


**Fig. S4 BiFC identifies that NHL3 termini are both cytosolic facing**

Maximum projections of confocal z-stacks of *N. benthamiana* epidermal cells transiently expressing split YFP constructs. The construct combinations are indicated above in each image panel, and fluorescence of split-YFP constructs is shown in yellow with chlorophyll autofluorescence in magenta. All scale bars are 25 µM.

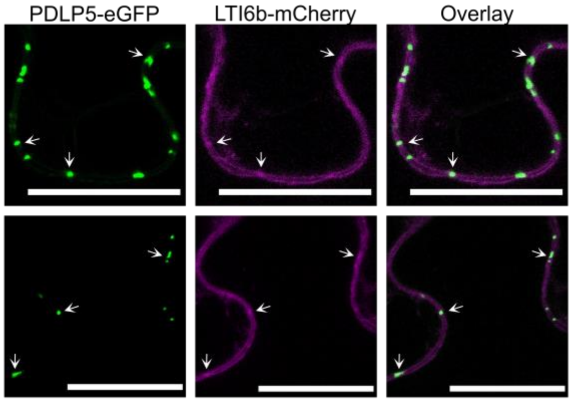


**Fig. S5 Transient expression of *pACT2::LTI6b-mCherry* identifies LTI6b-mCherry fluorescence co-incident with plasmodesmata**

Confocal micrographs of *N. benthamiana* transiently expressing *PDLP5-eGFP* and *pACT2::LTI6b-mCherry*. PDLP5-eGFP is in green and LTI6b-mCherry is in magenta. Arrows indicate co-localization at plasmodesmata. Scale bar is 25 µm.

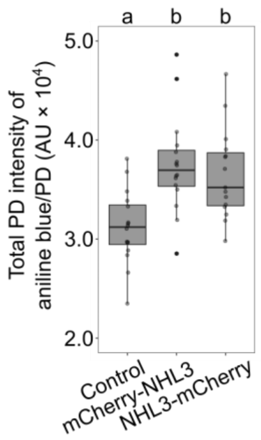


**Fig. S6 mCherry-NHL3 functions similarly to NHL3-mCherry when transiently expressed in *N. benthamiana***

Quantification of aniline blue stained plasmodesmata (PD)-associated callose denoted by arbitrary units (AU) using automated image analysis in *N. benthamiana* transiently expressing p19 only (Control), mCherry-NHL3 or NHL3-mCherry. Three to four z-stack image series for four biological replicates taken per construct combination. Independent factor 'Gene Expressed' was determined as significant (ANOVA: $F = 13.0$, $df = 2$, $p<0.0001$), with significant differences denoted by a and b, $p<0.001$.

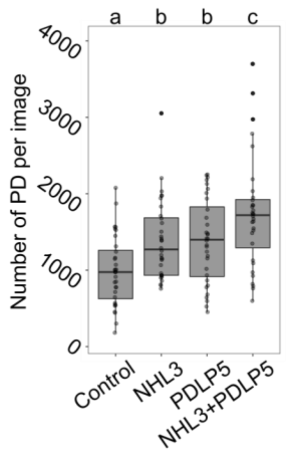


**Fig. S7 Combined expression of NHL3-mCherry and PDLP5-eGFP (NHL3+PDLP5) increases the number of plasmodesmal callose deposits detected per image**

Number of aniline blue stained plasmodesmata (PD)-associated callose using automated image analysis in *N. benthamiana* transiently expressing p19 only (Control), NHL3-mCherry (NHL3), PDLP5-eGFP (PDLP5), or NHL3-mCherry and PDLP5-eGFP (NHL3+PDLP5). Combined data from two independent experiments, with four z-stack image series from four biological replicates taken per construct combination in each experiment (for a total of 32 images per construct combination). Independent factor 'Gene Expressed' was determined as significant (ANOVA: F = 16.2, df = 3, $p$<0.0001), with significant differences denoted by a, b and c, $p$<0.01.

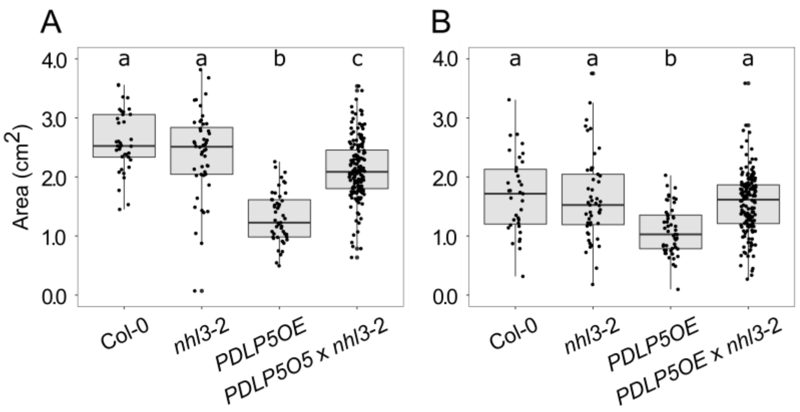


**Fig. S8 Whole rosette area of 21-day old Col-0, *nhl3*-2, *PDLP5OE* and *PDLP5OE* × *nhl3*-2**

Two independent experiments show A) partial or B) whole reversion of *PDLP5OE* dwarf phenotype. (A) was analyzed by a linear mixed-effects model with random effect being the different individual plates plants were grown. Independent factor 'Genotype' was determined as significant (ANOVA: F = 61.8, df = 3, $p<0.0001$), with significant differences denoted by a, b and c, $p<0.0001$. For Col-0, *nhl3*-2, *PDLP5OE* and *PDLP5OE* × *nhl3*-2, n = 37, 48, 49 and 160 respectively. For (B), a linear mixed-effects model was used with random effects being the different individual plates plants were grown. Independent factor 'Genotype' was determined as significant (ANOVA: F = 24.5, df = 3, $p<0.0001$), with significant differences denoted by a and b, $p<0.0001$. For Col-0, *nhl3*-2, *PDLP5OE* and *PDLP5OE* × *nhl3*-2, n = 35, 49, 51 and 158 respectively.

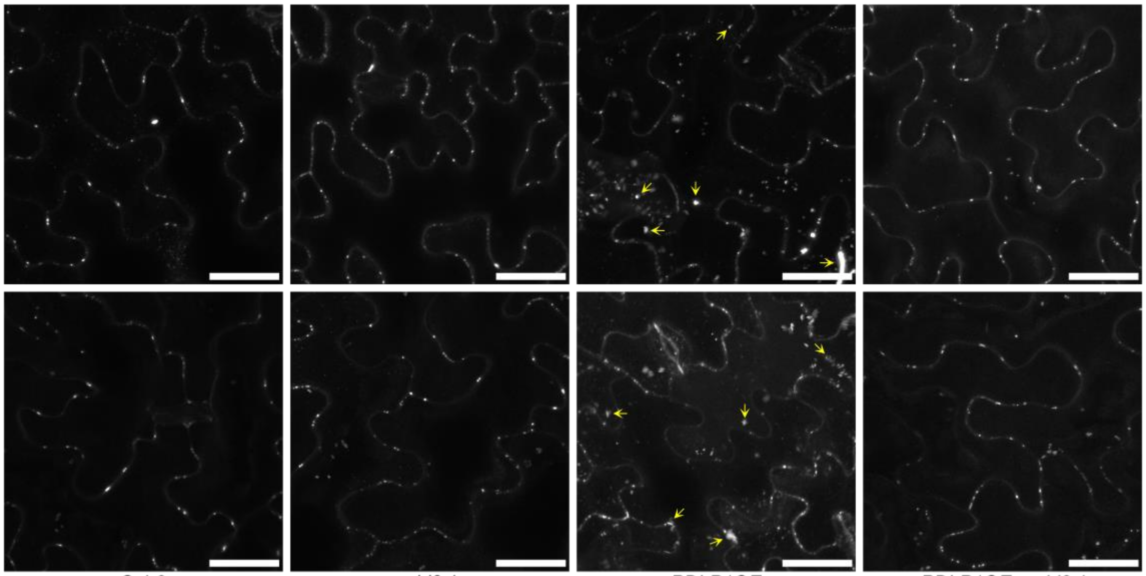


**Fig. S9 Callose staining of Col-0, *nhl3*-1, *PDLP1OE* and *PDLP1OE* × *nhl3*-1**

Representative pictures of callose staining per genotype with non-plasmodesmal callose indicated by white arrows. All scale bars are 25 µM.

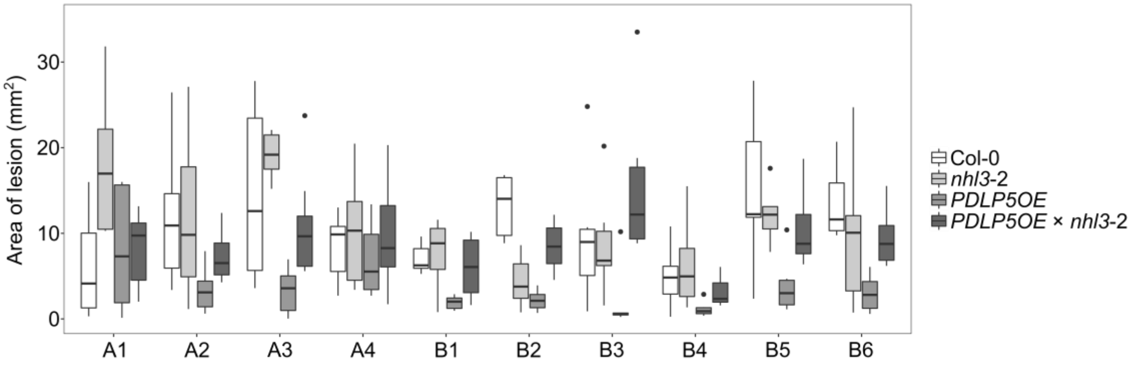


**Fig. S10 Average area of *Botrytis cinerea* disease lesions for biological replicates of Col-0, *nhl3*-2, *PDLP5OE* and *PDLP5OE* × *nhl3*-2**

Individual lesions (2 dpi) plotted per genotype for a given experimental plate. Plates had three or four leaves per genotype per plate. Plates denoted A and B were prepared on different days.

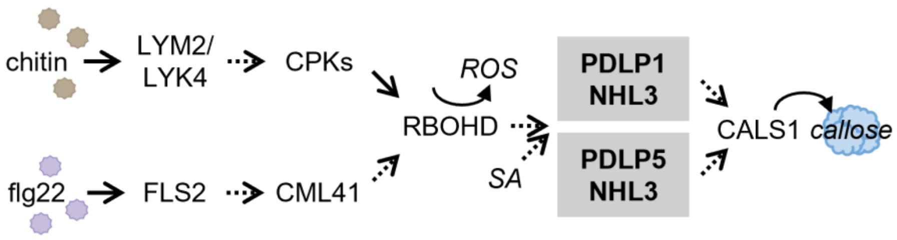


**Fig. S11 Alternative model of elicitor induced plasmodesmal closure**

MAMP elicitors chitin and flg22 activate different receptor complexes that signal via specific pathways to RBOHD as per Fig. 4G. RBOHD produces ROS that transmit plasmodesmal signals downstream, converging with SA elicited signals at a either a PDLP1-NHL3 or PDLP5-NHL3 complex that integrates the information and activates CALS1 to produce callose and close plasmodesmata. Established paths are indicated by solid arrows and yet to be understood paths are indicated by dashed arrows.

**Table S1 PDLP5 prey hit candidates cross-referenced with *Arabidopsis* plasmodesmal proteomes**

Split Ubiquitin Hits indicates how many protein fragments were detected in the screen. + indicates candidates found in the *Arabidopsis* Plasmodesmal Proteome (Fernandez-Calvino et al., 2011 (5)). The PD/PM ratio was taken from Brault *et al*., (2019 (6)) where its relative enrichment in the plasmodesmata fraction was compared to contaminant fractions (*i.e*., the PM, microsomal, total cell and cell wall fractions).

| Gene Identifier | Split Ubiquitin Hits | Plasmodesmal Proteome | PD/PM ratio | Name |
|---|---|---|---|---|
| **AT5G06320** | **1** | **+** | **48** | **NHL3** |
| AT1G71695 | 1 | + | | peroxidase |
| AT1G20330 | 1 | + | | SMT2 |
| AT4G23630 | 1 | + | | RTN1 |
| AT4G25810 | 1 | | 816 | XTR6 |
| AT4G35100 | 9 | + | | PIP3 |
| AT2G37170 | 3 | + | | PIP2B |
| AT1G11260 | 1 | + | | STP1 |
| AT1G53210 | 1 | + | | NCL |
| AT2G01470 | 1 | + | | STL2P |
| AT3G53420 | 1 | + | | PIP2A |
| AT3G54140 | 1 | + | | NPF8.1 |
| AT4G30190 | 1 | + | | AHA2 |

**SI References**

1. B. Castel, L. Tomlinson, F. Locci, Y. Yang, J. D. G. Jones, Optimization of T-DNA architecture for Cas9-mediated mutagenesis in Arabidopsis. *PLoS One* **14**, e0204778 (2019).
2. C. Engler, *et al.*, A Golden Gate modular cloning toolbox for plants. *ACS Synth. Biol.* **3**, 839–843 (2014).
3. M. G. Johnston, C. Faulkner, A bootstrap approach is a superior statistical method for the comparison of non-normal data with differing variances. *New Phytol.* **230**, 23–26 (2021).
4. E. E. Tee, M. G. Johnston, C. Faulkner, faulknerfalcons/Tee-etal-2023. Zenodo. https:/doi.org/10.5281/ZENODO.7680790. Depositied 27 February 2023.
5. L. Fernandez-Calvino, *et al.*, Arabidopsis Plasmodesmal Proteome. *PLoS One* **6**, e18880 (2011).
6. M. L. Brault, *et al.*, Multiple C2 domains and transmembrane region proteins (MCTPs) tether membranes at plasmodesmata. *EMBO Rep.* **20**, e47182 (2019).
